# Phenotypic and genotypic features of first biofilm forming nasopharyngeal colonized *Streptococcus pneumoniae* isolates

**Published:** 2017-08

**Authors:** Seyed Fazlollah Mousavi, Bahman Mirzaei, Bahareh Shaghaghi, Pantea Jalali, Tahereh Setayesh, Seyed Hamid Moosavi

**Affiliations:** Department of Bacteriology & Microbiology Research Center, Pasteur Institute of Iran, Tehran, Iran

**Keywords:** Biofilm, *Streptococcus pneumoniae*, PFGE, Clonal diversity

## Abstract

**Background and Objectives:**

Designing control and therapeutic policies for antibiotic resistant *Streptococcus pneumoniae*, which is an important causative agent of several invasive and noninvasive infectious diseases and its carriage rates, has been described as the main target in World Health Organization (WHO). The present study was conducted to determine antibiotic resistance pattern, evaluate biofilm forming ability in *S. pneumoniae* isolates, and find the genetic relationship between cultured strains.

**Materials and Methods:**

Following the isolation and identification of *S. pneumoniae* strains from nasopharyngeal swabs, the ability of biofilm formation and susceptibility pattern of the isolates were screened using semi-quantitative microplate and disk diffusion procedures. Subsequently, Pulse field gel electrophoresis (PFGE) method was used to determine the clonal diversity of isolates.

**Results:**

The pneumococcal colonization rate in this study was found to be 24%. A large number of our isolates had strong biofilm forming ability. However, there was variation in antibiotic resistance patterns of isolates in children who lived in nursery houses. The genetic similarity among the isolates in PFGE varied from 26.5% to 100% in our isolates. This was the first report of biofilm formation of nasopharyngeal colonized *S. pneumoniae* in Iran. Genetic variations were also noticeable, when the isolates were fingerprinted by PFGE.

**Conclusion:**

The findings of this study revealed the need for thoughtful use of antimicrobial agents, continued monitoring of pneumococcal resistance patterns, and prevention of the spread of multi-drug resistant clones.

## INTRODUCTION

*S. pneumoniae* is the leading cause of community-acquired pneumonia, meningitis and otitis media in children. It may colonize up to 60% of children, and the disease occurs under certain conditions (1). The incidence of disease may be underestimated due to the widespread adoption of antibiotics and technical failure in culturing the organism (2). Nasopharyngeal carriage of antibiotic resistant *S. pneumoniae* is a sign of the prevalence of resistant strains in a population, and thus has been used to measure the resistance of *S. pneumoniae* in various communities (2). Development of antibiotic resistant strains causing nasopharyngeal colonization and infections varies with age, geographic location and socioeconomic status (3).

The carrier state is a prerequisite for the invasion of the bloodstream or spread of the microorganism to other organs. In the carrier state, *S. pneumoniae* survives in a sessile mode of growth for prolonged intervals of time.

The oral streptococci including *Streptococcus gordonii*, *S. mutans* and *S. intermedius* provided information on the possible behavior of pneumococci in different environments and situations, particularly planktonic growth vs. sessile growth in the biofilm. In this group of bacteria, formation of biofilm is linked to competence (4).

The emergence of drug-resistant strains of *S. pneumoniae* has become a major concern in antimicrobial treatment of such infections (5). Therefore, different epidemiological markers are required to survey *S. pneumoniae* infections; moreover, phenotypic and genotypic schemes have been developed to assist in epidemiological investigations. Pulsed field gel electrophoresis (PFGE) is one of the most commonly used methods for *S. pneumoniae* molecular typing (6). It holds a notorious discriminatory ability, high reproducibility and fully-determined interpretative criteria, and it has been recognized as one of the main tools for epidemiological and surveillance studies (7).

This study was designed to determine the nasopharyngeal carriage rate of *S. pneumoniae* among children at 4 nursery houses of Tehran and find antibiotic resistance patterns of isolates. Moreover, the potential of isolates in biofilm formation was checked to find whether there were any correlations between the strength of biofilm formation and various pulse types obtained by PFGE.

## MATERIALS AND METHODS

### Strain isolation and identification.

Isolates were cultured from 317 nasopharyngeal swabs of children who lived in 4 different nursery centers (Center 1: Ameneh, Center 2: Shobayr, Center 3: Turkamani, and Center 4: Hazrat-e-Roghayeh) during November 2014 to March 2015. Isolated strains were identified using the conventional microbial tests according to the scheme described earlier (8). These centers are located in 4 different regions of Tehran, Iran. Nasopharyngeal swab samples were taken with rayon-tipped, flexible, aluminum-shaft swabs (Medical Wire and Equipment Company, Town, United Kingdom), according to World Health Organization guideline (8, 9). Briefly, the swabs were inserted into 3 mL of Stuart Transport Medium (BD; Becton, Dickinson and company, Sparks, MD, USA), which was transported to the Pasteur Institute Microbiology Department immediately.

### PCR for detection of *cpsA* gene.

Cultured strains were confirmed to determine gene encode polysaccharide capsule (*cpsA*) using specific primers (10).

**Fig. 1.**
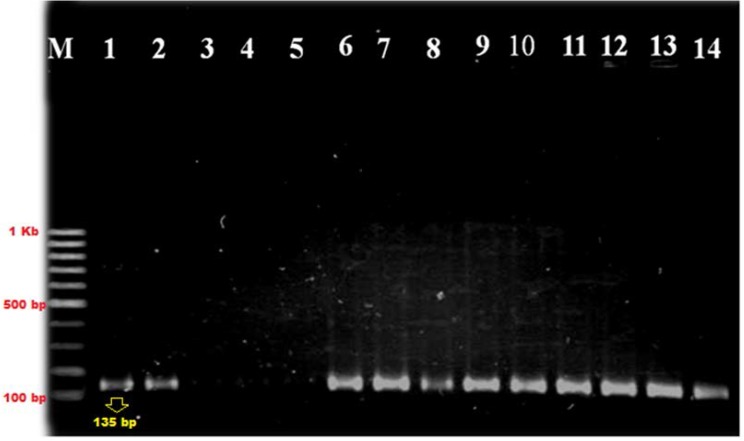
Molecular identification of *S. Pneumoniae* isolates by *cpsA* gene M, 100bp DNA ladder; Lane 1, Control strain (*S. pneumonia* ATCC6305); Lane 2- 14alpha–hemolytic and optochin susceptible isolated strain

PCR amplifications were performed in the final volume of a 25 μL PCR mixture, containing 12 μL supernatant, 1x PCR buffer, 1.5 mM MgCl_2_, 0.2 mM each of dNTPs, 0.5 U Taq DNA polymerase (HT Biotechnology, Cambridge, UK), and each primer (5 pmol). PCR reaction consisted of denaturation at 94°C for 2 minutes, annealing at 56°C for 30 seconds, and extension at 72°C for 2 minutes, followed by 29 cycles. The amplicons (130 bp) were analyzed by electrophoresis on a 1% agarose gel and visualized using UV transilluminator after staining with ethidium bromide (Fig. 1).

### Antibiotic Susceptibility Testing.

Susceptibility of isolates to antibiotics was assessed using Disks (BD BBLTM Sensi DiscTM) impregnated with amoxicillin-clavulanic acid (20 + 10μg), tetracycline (30 μg), tobramycin (10 μg), bacitracin (10 U), nalidixic acid (30 μg), gentamicin (10 μg), erythromycin (15 μg), oxacillin (1μg), azithromycin (15mg), and rifampin (5 μg), according to the clinical laboratory standard institute guideline (11). *S. pneumoniae* ATCC 49619 and *S. pneumoniae* ATCC 6301 were used as controls. Biofilm formation was tested as previously described (12), with slight modifications. Briefly, pneumococcal strains were streaked on sheep blood agar plates and grown for 18 to 24 hours in 5% CO_2_ at 37°C. Bacterial suspensions in Todd Hewitt broth with 1% glucose were prepared at OD_600_= 0.5 and used to inoculate 96-well polystyrene microplates. Plates were incubated for 18 hours in 5% CO_2_ modified atmosphere. After incubation, growth was recorded by reading the absorbance at OD_600_ (Biotech, USA); the supernatant was discarded, and plates were gently washed with saline solution, air dried, and stained with Hucker’s crystal violet. Excess stain was washed off with tap water. The optical density of the biofilm was measured at 570 nm in an automatic spectrophotometer (Biotech, USA). The process was done in duplicate form for each isolate (1).

*S. pneumoniae* ATCC 6303 were used as a control strain to determine the optimal conditions for biofilm formation (1).

### Pulse field gel electrophoresis (PFGE).

For molecular typing of *S. pneumoniae* strains, PFGE was used as described previously (13). In brief, the plugs were digested overnight with *SmaI* (Boehringer Mannheim), according to the manufacturer’s instructions. DNA fragments were separated using the CHEF DR-III apparatus (Bio-Rad, Birmingham, UK). Electrophoresis was performed for 20 hours using linearly ramped pulse times, beginning with 0.2 s and ending with 25 s, at an applied voltage of 6 V/cm at 14°C.

**Fig. 2.**
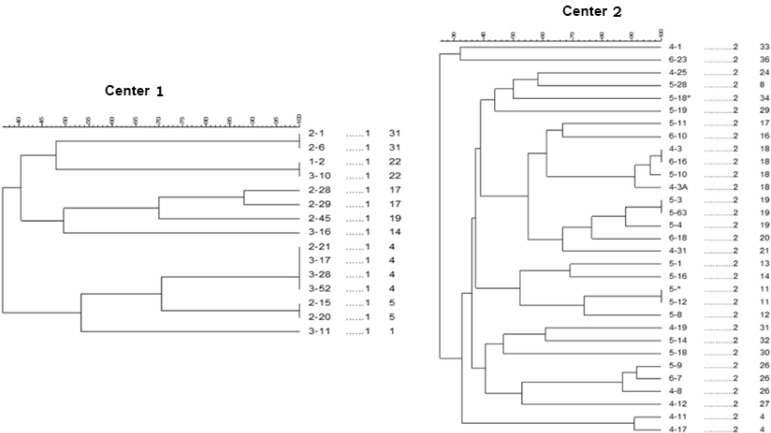
Dendrogram of 8 (Center 1) and 22 (Center 2) Pulsed field fel electrophoresis profiles from 15 and 31 *S. pneumoniae* isolates

**Fig. 3.**
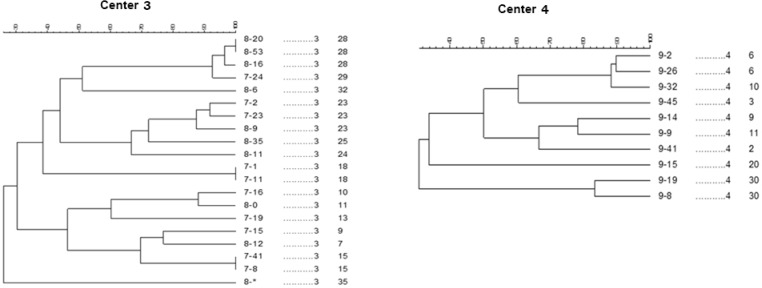
Dendrogram of 14 (Center 3) and 8 (Center 4) Pulsed field gel electrophoresis profiles from 20 and 10 *S. pneumoniae* isolates

*Salmonella choleraesuis* serotype Brander up H9812 was used as a molecular size marker. The DNA banding patterns were analyzed using Gel-Compare II software (Applied math NV, St-Martens-Latem Belgium). PFGE pattern was compared and interpreted according to criteria of Tenover et al. (14) (Figs. 2, 3).

### Statistical analysis.

Statistical analysis was performed using SPSS Version 16.0 (SPSS Inc., Chicago, IL, USA). Statistical significance was assessed using analysis of variance for repeated measures (OD values). In case of statistically significant results, different tests were compared in pairs according to pre-test hypothesis using a t test for paired samples (OD values).

## RESULTS

### Strain collection.

A total of 76 (24%) isolates of *S. pneumoniae* were identified after biochemical and PCR methods. A total of 41 males (54%) and 35 females (46%) children were culture- positive for *S. pneumoniae*.

None of the children had been vaccinated with pneumococcal vaccines before. No child had been hospitalized in the 14 days prior to sampling.

### Carriage rate.

All 317 nasopharyngeal swab samples collected from nursery houses were located in 4 different regions of Tehran (Center 1, Center 2, Center 3 and Center 4). Carriage rates were 15.4%, 30%, 28.5% and 28.5% for Centers 1 to 4, respectively.

### Antibiotic susceptibility testing.

All *S. pneumoniae* isolates were resistant to at least 3 out of 10 antibiotics. All isolates that were identified as penicillin (oxacillin) and nalidixic acid resistant were susceptible to bacitracin and rifampicin (Table 1). Antibiotic susceptibility showed different patterns in different centers. Isolates from Centers 1 and 3 showed more resistance to different antibiotics compared to isolates of other centers; isolates of Center 2 showed less resistance to different antibiotics compared to isolates of other centers.

**Table 1.** Susceptibility of *S. pneumoniae* isolates to various antibiotics obtained by disk diffusion method

**Antibiotics tested**	**High resistance (%)**	**Intermediate resistance (%)**	**Sensitive (%)**
Tetracycline (30μg)	5	14	81
Bacitracin (10U)	-	-	100
amoxicillin-clavulanic acid (20+10 μg)	26	-	74
Erythromycin (15μg)	86	-	14
Gentamycin (10μg)	94	1.3	4.7
Nalidixic Acid (30μg)	100	-	-
Tobramycin (10μg)	98.7	-	1.3
Azithromycin (15μg)	86	-	14
Oxacillin (1μg)	100	-	-
Rifampicin (5μg)	-	-	100

### Biofilm formation.

Different capabilities of biofilm formation from *S. pneumoniae* isolates were observed. A baseline calculation of 3 standard deviations revealed none, weak, and strong biofilm formers. Non-biofilm formers are considered as 0.023 above the mean OD = 0.050 of a clean tissue culture plate stained by the OD = 0.120 as the threshold below. This way, only 5 strains out of 75 were found to be non-biofilm formers. Five isolates could be classified as weak biofilm formers (0.120<OD<0.240), while the other strains tested were strong biofilm producers (66 isolates).

**Fig. 4.**
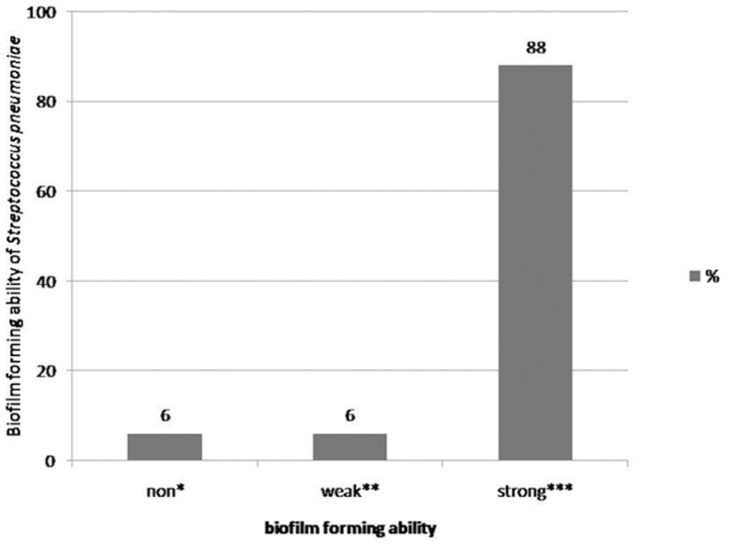
Biofilm forming ability of all isolates,*Non-biofilm former: The strain does not have any ability to form biofilm. **Weak-Biofilm Former: The strain has a weak ability to form biofilm (0.120<OD<0.240), ***Strong- Biofilm Former: The strain has strong ability to form biofilm.

Biofilm formation ability patterns appeared differently in different centers (Fig. 4).

Differences in susceptibility results were not observed when we compared biofilm and non-biofilm-forming *S. pneumoniae* isolates.

### PFGE analysis.

All isolates referred to PFGE were compared by Dice correlation coefficient with 85% cutoff and 1% tolerance using GelCompare II software (Applied Math NV, St-Martens- Latem Belgium).

The percent of similarity varied from 26.3% to 100% among the isolates by the Dice coefficient. In total, 36 different PFGE types were identified from 4 separate centers. Center 1 was heterogeneous, as 8 specific banding patterns were found for 15 isolates. More diversity was found among the isolates at Centers 2 (n = 31), 3 (n = 20) and 4 (n = 10), as 22, 14, and 8 patterns were found in the mentioned centers, respectively. A total of 13 isolates were considered individual isolates, presenting less than 80% intraclonal similarity. The pulse Types 4 (n = 6), 18 (n = 6), 11 (n = 4) and 19 (n = 4) were the dominant types found in the 4 centers.

## DISCUSSION

The nasopharyngeal carriage of *S. pneumoniae* is widely prevalent in young children and has been related to the spread of the pathogen and development of disease (15). Furthermore, antibiotic-resistant *S. pneumoniae* nasopharyngeal colonization has increased over the last decade (16). In this study, nasopharyngeal carriage rate of *S. pneumoniae* in children was 24%. Pneumococcal nasopharyngeal carriage studies conducted on children showed a prevalence of pneumococcal carriage to be 19% to 43% (3). The highest nasopharyngeal colonization rates were reported in Africa (85%–87.2%) (17).

In the present study, various carriage rates of *S. pneumoniae* were identified. The highest rate was observed in Center 2 and the lowest in Center 1 in Tehran. Centers 3 and 4 had similar carriage rates. Although these children lived together in nursery houses (centers), our results revealed a variety of different antibiotic- resistant patterns in the isolates. These various patterns were detected in serotypes and DNA fingerprinting using PFGE method. Our study revealed that the incidence of penicillin-resistant strains among Iranian isolates is alarmingly high. The rate of resistance to penicillin in our isolates (100%) was higher than that reported previously in Iran (68%) (18) and other countries (19, 20). Penicillin resistance is also a matter of concern in some other countries like Spain, where the rate of resistance to penicillin has been reported to be more than 50% (21). Investigations in other countries have also indicated an increase in the prevalence of resistance to penicillin and other agents among *S. pneumoniae* strains (20, 22). The results of a study in Hong Kong revealed that 84.6% of the *S. pneumoniae* isolates cultured from nasopharyngeal swab samples of children who attend day care centers were resistant to penicillin (23). Non-susceptibility to penicillin among *S. pneumoniae* isolates was lower (86%) in hospitalized patients in 2003 compared to our data from healthy children (100%). This emphasizes the pressing warning of antibiotic resistance.

It seems that carriage of *S. pneumoniae* has been associated with occurrence of clinical diseases; moreover, the antibiotic susceptibility pattern of isolates reflects the susceptibility pattern of invasive strains.

The ability of upper respiratory pathogens including *S. pneumoniae* to persist in the nasopharynx and cause chronic diseases upon appropriate conditions may be associated with the ability to form a biofilm on mucosal epithelium (24). *S. pneumoniae* biofilm formation has been shown to occur in humans during nasopharyngeal colonization and recurrent otitis media. Pneumococcal biofilms have been detected in human sinus mucosa biopsies and resected adenoids from individuals with tonsillitis; moreover, biofilms have been observed within tympanostomy tubes collected from children with chronic otitis media (25). Fulfilling Koch’s postulates, biofilms, and biofilm-like pneumococcal aggregates have been observed in the middle ears of experimentally infected chinchillas as well as bronchial and nasal lavage fluids taken from the nasopharynx of infected mice, respectively (26, 27). A large variability in the amount of biofilm was observed among the strains examined; in any case, this appeared to be a very common feature among *S. pneumoniae*, as virtually all isolates were able to form some degree of biofilm. Such observation correlates well with the types of infections caused by this micro-organism (ie, otitis, meningitis, etc.), which have often been associated with the ability to form biofilm (28). We have seen nearly similar biofilm forming ability at different centers (Fig. 2). In agreement with other studies, differences in MICs values were not observed when we compared biofilm and non-biofilm-forming *S. pneumoniae* isolates. However, antibiotic resistance rates in biofilm forming clones exhibited different trends depending on their origin (13).

Using PFGE, 36 distinct pulse types were identified, of which 11 comprised a single isolate, and the remainder were clusters containing 2 to 6 isolates; they were found in the nasopharynx of children who lived in centers in Tehran. Our results revealed a large diversity in pulse types in each center. The percentage of genetic similarity among the isolates in PFGE varied from 26.5% to 100% in our isolates.

PFGE analysis revealed that most of identical PFGE patterns were found in the same cities, and mostly in the same childcare institutions. In contrast to the hypothesis that crowdedness in centers and the presence of children for 24 hours a day are major factors facilitating clonal spread, in this study, we identified a large clonal diversity in isolates of each orphanage. In spite of the condition of these children, their close contacts and living closely together in centers, we have seen several colons in each center. Some shared PFGE patterns were identified in centers, which might be the common types of *S. pneumoniae* in the community. The risk of invasive *S. pneumoniae* infections is greatly increased for children living in closed communities compared to those children cared for at home or in small family-operated day care centers, at least during the first 2 years of life (29). Although our data lacked information about day care center attendance and other aspects of lifestyle of other children, one can speculate that they represent a more diverse and wider sample of children living in the community, with a lower proportion of day care center attendees among them.

In conclusion, pneumococcal colonization rate of 24% was found in children although these children were living together in nursery houses (centers). Moreover, our results revealed a variety of different antibiotic resistant patterns and a large diversity in PFGE types in *S. pneumoniae* isolates. To our knowledge, this was the first report on biofilm formation of nasopharyngeal colonized *S. pneumonias* in Iran. The currency of the multidrug resistant phenotype poses a serious public health affair for increased treatment failure in the use of any drugs. The findings of this study emphasize the need for the thoughtful use of antimicrobial agents, continued monitoring of pneumococcal resistance patterns, and prevention of the spread of multi-drug resistant clones.
